# Get the ball rolling: update and perspective on the role of chloroplast plastoglobule-associated proteins under abiotic stress

**DOI:** 10.1093/jxb/eraf011

**Published:** 2025-01-11

**Authors:** Sheng Ying

**Affiliations:** Department of Biochemistry and Molecular Biology, Michigan State University, East Lansing, Michigan 48823, USA; RIKEN Center for Sustainable Resource Science, Japan

**Keywords:** Abiotic stress, chloroplast, phosphorylation, plastoglobules, proteome, root, transcriptome

## Abstract

Plastid-localized plastoglobules (PGs) are monolayer lipid droplets typically associated with the outer envelope of thylakoid membranes in chloroplasts. The size and number of PGs can vary significantly in response to different environmental stimuli. Since the early 21st century, a variety of proteins attached to the surface of PGs have been identified and experimentally characterized using advanced biotechnological techniques, revealing their biological functions. This article aims to assess the latest discoveries regarding PG-associated proteins and explore their dynamics under both single and combined abiotic stress conditions, providing insights into the critical role of plastid lipid droplets in plant adaptation to global climate-related challenges.

PGs are representative lipid droplets found in various types of plastids, including chloroplasts, chromoplasts, and leucoplasts. In chloroplasts, PGs are contiguous with the outer lipid leaflet of the thylakoid membrane, and their size and number are closely linked to the state of the thylakoid membrane system (van Wijk and Kessler, 2017). For instance, during senescence, the dismantling of thylakoid membranes leads to the formation of larger PGs. The monolayer surface of PGs is composed primarily of galactolipids, such as monogalactosyldiacylglycerol (MGDG) and digalactosyldiacylglycerol (DGDG), while the core is enriched in neutral lipids such as triacylglycerols (TAGs) and various prenyl-lipid species, which are involved in photosynthesis and photoprotection.

Recent proteomic advances have identified ~30 conserved proteins, termed PG core proteins, across several species, including Arabidopsis, maize, and tomato. Studies on these proteins in Arabidopsis reveal their roles in senescence, chloroplast biogenesis, carotenoid metabolism, and redox regulation. These proteins are crucial for lipid metabolism, developmental transitions, and environmental adaptation (Box 1) (van Wijk and Kessler, 2017; Shanmugabalaji *et al.*, 2022). For instance, tocopherol cyclase (VTE1) is essential in maintaining lipophilic antioxidants balance, thus protecting the thylakoid from photooxidative damage by scavenging reactive oxygen species (ROS) (Porfirova *et al.*, 2002; Vidi *et al.*, 2006).

Box 1. **Illustration of the functions of plastoglobule core proteins**

Environmental stimuli (i.e. high light stress, etc.) or developmental clues (i.e. senescence) induce PG formation at the margin of thylakoid membrane. Diverse lipids and metabolites are stored in the PG to maintain thylakoid homeostasis under unfavorable conditions. Approximately 30 PG core proteins, which are studded on the surface of the PG, are identified through advanced proteomic approaches. However, only a limited number of members have been functionally and genetically investigated (van Wijk and Kessler, 2017; Kim and Kim, 2022; Shanmugabalaji *et al*., 2022). The functions of a representative or recently characterized PG core protein are illustrated in the diagram. ① During leaf senescence, chlorophyll is gradually degraded into phytol. PES1 and PES2 combine phytol and free fatty acids into fatty acid phytyl esters. In addition, PES1 and PES2 catalyze the biosynthesis of TAG from DAG and free fatty acids (Lippold *et al*., 2012). ② FBN1a, 1b, and 2 are the most abundant structural PG proteins. They interact with each other to form homo- or heterodimers, or hetero- oligomers. The FBN-based interaction provides a framework to ensure the formation of PG protein complexes, such as ①, and ④–⑧ (Gámez-Arjona *et al*., 2014a, b; Torres-Romero *et al*., 2021). ③ PG-localized NDC1 catalyzes the reduction of PQ-9 to PQ-9-H2. Then, VTE1 converts the reduced substrate PQ-9-H2 into PC-8. The activity of VTE1 might be activated by the phosphorylation of ABC1K1 and 3 (Porfirova *et al*., 2002; Vidi *et al*., 2006; Eugeni Piller *et al*., 2011; Lundquist *et al*., 2013). ④ Starch synthase 4 (SS4) could interact with FBN1a and b and other unknown PG-localized protein, affecting the production of chloroplast starch granules (Gámez-Arjona *et al*., 2014b). ⑤ In melon fruit, FBN1 interacts with ORANGE (OR) to enhance its stability. The increased structural FBN1 stimulate PG proliferation and thereby carotenoid accumulation (Zhou *et al*., 2023). ⑥ In the rice chloroplast, FBN7 interacts with and stabilizes two β-ketoacyl-ACP synthase I (KAS Ia/b), resulting in the production of small PG clusters at the surface of existing PGs. Notably, OsFBN7 I studded on the PG surface to prevent the coalescence and aggregation of smaller PGs (Li *et al*., 2023). ⑦ CCD4 cleaves carotenoid substrates (i.e. β-carotene, lutein, and zeaxanthin) into different colorless compounds (i.e. β-ionone, β-cyclocitral, and β-citraurin). In addition, the activity of CCD4 might be regulated by the phosphorylation of ABC1K1 and 3. Recently, a co-immunoprecipitation (Co-IP) analysis revealed that CCD4 could directly interact with FBN2 (Lundquist *et al*., 2013; Rottet *et al*., 2016; Torres-Romero *et al*., 2021). ⑧ During senescence, the PG-localized FBN2 recruits enzymes of jasmonic acid (JA) biosynthesis (i.e. LOX3, LOX4, AOC, and AOS). The complex facilitates the production of JA and recycling of thylakoid dissembled lipids (Kim *et al*., 2022; Lundquist *et al*., 2013; Roberto *et al*., 2021; Torres-Romero *et al*., 2021).

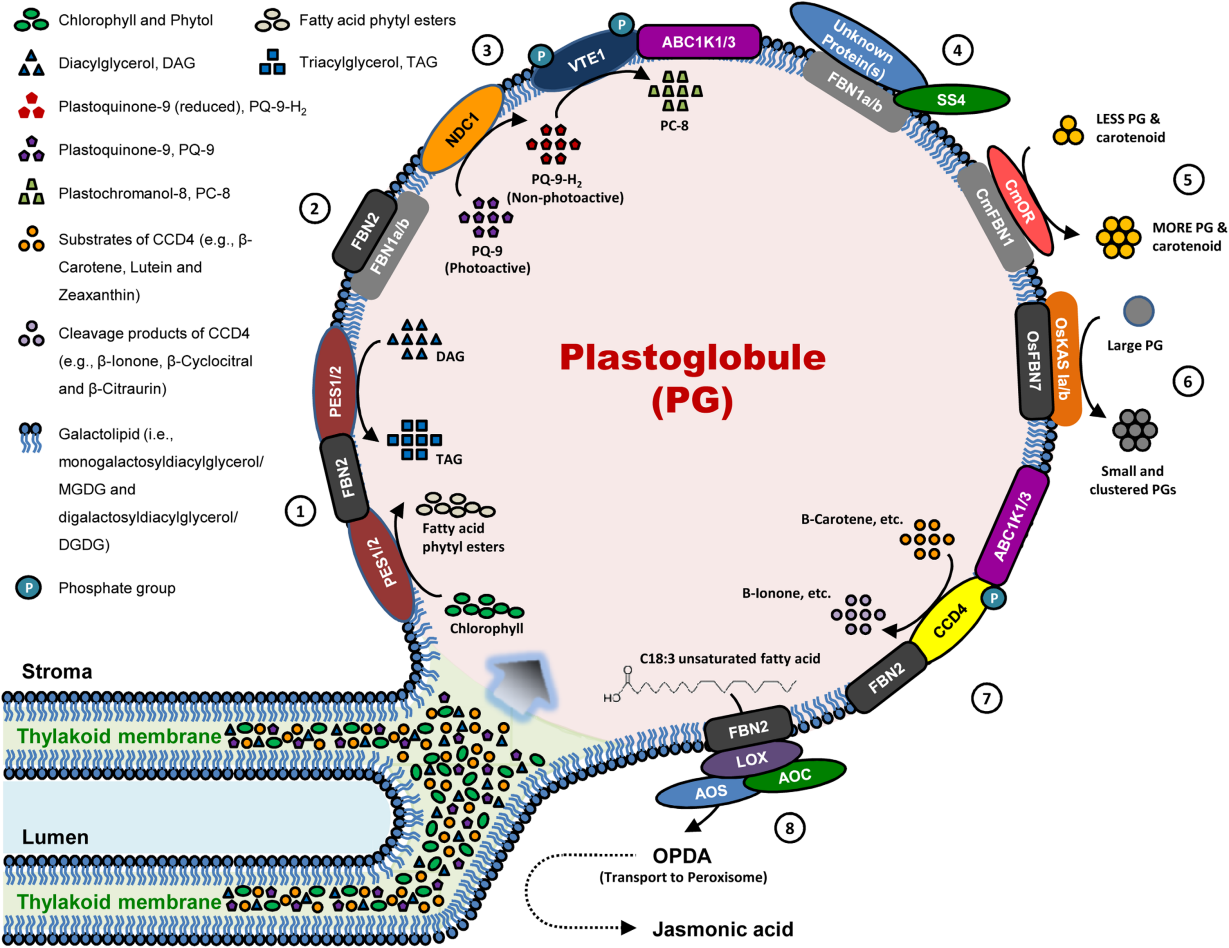

## Involvement of PG core proteins in abiotic stress responses

Various abiotic stresses, such as nitrogen deprivation, senescence, and high light stress, trigger the breakdown of thylakoid membranes, leading to an increase in the size of PGs to store the disintegrated lipids and chlorophyll. As a result, the formation of larger PGs serves as an ultrastructural marker for assessing leaf stress symptoms (Zechmann, 2019). Through the functional characterization of PG-associated proteins, we are gradually uncovering the crucial roles of PGs in responding to environmental stress (Shanmugabalaji *et al.*, 2022). For instance, during leaf senescence in Arabidopsis, PES1 and PES2 proteins function as acyltransferases and play a positive role in TAG accumulation in PGs (Lippold *et al.*, 2012). Tocopherol cyclase, due to its role in preventing lipid peroxidation, has been shown to actively contribute to abiotic stress responses (Allu *et al.*, 2017; Surówka *et al.*, 2020).

Fibrillins (FBNs) are the most abundant proteins in the PG proteome, accounting for >50%. These proteins not only contribute structurally to PG formation but also recruit other PG-associated or unknown proteins and enzymes to form complexes that facilitate various biochemical processes (Box 1). The FBN family has been studied in multiple plant species and shown to play a role in abiotic stress responses (Kim and Kim, 2022). For example, manipulation of the *OsFBN1* gene in rice affected PG formation and jasmonic acid (JA) synthesis during heat stress (Li *et al.*, 2019). In Arabidopsis, FBN2 protected the photosynthetic system from high light stress and inhibited JA-induced senescence (Kim *et al.*, 2022).

**Fig. 1.**
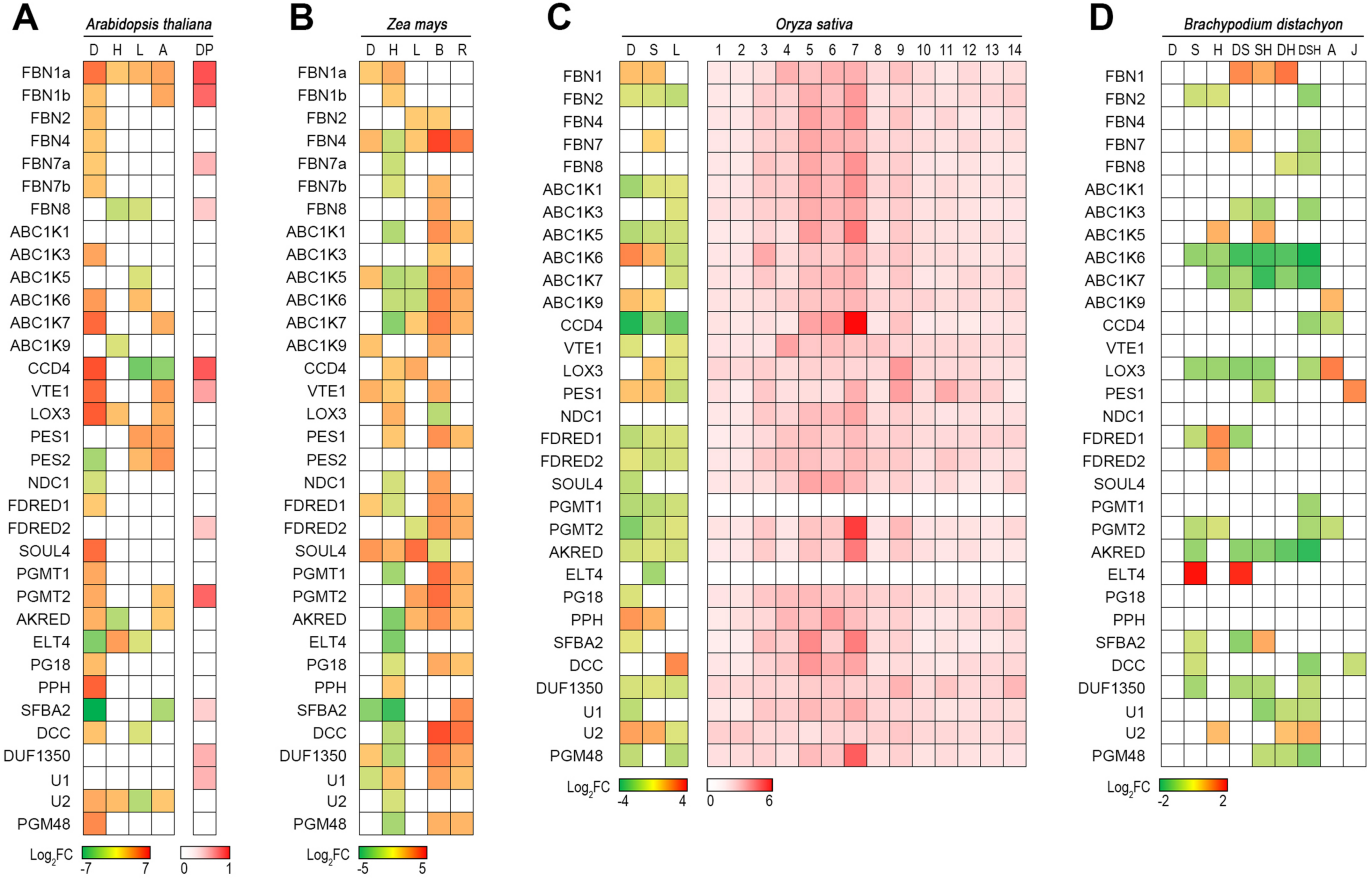
Transcriptional and proteomic analysis of plastoglobule (PG)-localized core genes. (A) Transcriptional (left panel) changes of Arabidopsis PG core genes during different abiotic stress (D, H, L, and A). Data are extracted from and analyzed based on the Expression Altas (https://www.ebi.ac.uk/gxa/home). Right panel, relative protein abundance changes of Arabidopsis PG core proteins under water-deficit stress (DP). Data are extracted from and analyzed based on Fulcher *et al*. (2024, Preprint). (B) Transcriptional response of maize PG core genes under different abiotic stress (D, H, L, B, and R). Data are extracted from and analyzed based on the Expression Altas (https://www.ebi.ac.uk/gxa/home). (C) Transcriptional (left panel) changes of rice PG core genes during different abiotic stress (D, S, L). Data are extracted from and analyzed based on ePlant (https://bar.utoronto.ca). Right panel, relative protein abundances of rice PG core proteins in different tissues. Data are extracted from Li *et al*. (2024). 1, root tip; 2, whole root; 3, culm and leaves; 4, stem; 5, leaf sheath; 6, auricle, ligule, and lamina joint; 7, leaf blade; 7, spike neck; 9, mature spikelet; 10, pistil; 11, pollen; 12, early-stage immature seeds; 13, grain-filling seeds; and 14, mature seeds. OsOGMT1 and OsELT4 were not detected in this study. (D) Transcriptional changes of *Brachypodium* PG core genes during different combinational abiotic stress treatments (D, S, H, DS, SH, DH, DSH, A, and J). Data are extracted from and analyzed based on Kakei *et al*. (2015) and Shaar-Moshe *et al*. (2017). D, drought; H, high temperature/heat; L, low temperature (4 °C); B, blue light; R, red light; S, salinity; DS, drought and salinity; SH, salinity and heat; DH, drought and heat; DSH, drought, salinity, and heat; A, ABA (100 μM for Arabidopsis; 10 μM for *Brachypodium*); and J, JA (30 μM). All data are subjected to statistical analysis, and significant differences (*P*adj <0.05) are colored in the heat maps.

ABC1Ks are atypical protein kinases found in both prokaryotes and eukaryotes. Six of the eight AtABC1Ks were identified in the PG proteome, where they interact with and phosphorylate other core proteins, indicating their involvement in various regulatory pathways (Fig. 1). Biochemical and genetic analyses have shown that AtABC1K1/PGR6 and AtABC1K3 play opposing roles in regulating plastoquinone homeostasis in response to light treatments (Huang *et al.*, 2015; Pralon *et al.*, 2020). The *AtABC1K7* gene participates in abscisic acid (ABA)-mediated oxidative stress responses (Manara *et al.*, 2016). In rice, *OsABC1K3* is highly expressed in young leaves and is essential for plant adaptation to high light stress (Chen *et al.*, 2023).

Other PG core proteins, previously categorized as ‘unknown’ proteins, have also been characterized experimentally. In Arabidopsis, the metallopeptidase PGM48 has been shown to positively regulate leaf senescence, possibly by degrading the enzyme CCD4 (Bhuiyan *et al.*, 2016). The *pg18* mutant exhibited a significant reduction in chlorophyll and carotenoids, but an increase in xanthophyll derivatives, especially under high light stress, suggesting an important role for PG18 in supporting the photosynthetic system (Espinoza-Corral *et al.*, 2019).

In summary, there is substantial evidence that chloroplast PG core proteins play critical roles in abiotic stress responses. Future studies should focus on manipulating the transcript levels or protein abundances of these proteins in economically important crops to mitigate stress symptoms and enhance photosynthetic efficiency.

## Dynamics of PG core genes/proteins under abiotic stress

Transcriptional analysis of PG core genes in four model species, namely Arabidopsis, maize, rice, and *Brachypodium distachyon*, has revealed their strong responses to various environmental stimuli (Fig. 1; Table 1; Supplementary Tables S1–S4). Drought stress and extreme temperatures induce the expression of 26 out of 34 and 13 out of 34 Arabidopsis PG genes, respectively. Except for *FBN8*, all PG-associated FBN genes are up-regulated by drought stress, reflecting their structural role in the formation of thylakoid-associated PGs. The phytohormone ABA, an intermediate in carotenoid metabolism in chloroplasts, influences PG-related genes. The PG-localized CCD4 enzyme, which is involved in β-carotene metabolism, is regulated in opposing directions by drought stress and ABA treatment, with its expression being up-regulated by drought and down-regulated by ABA. ABA also induces the expression of other PG genes involved in lipid metabolism. Additionally, FBNs have been reported to play a role in ABA-mediated photoprotection (Yang *et al.*, 2006). This suggests that ABA signaling might play a key role in fine-tuning PG production in response to stress. In maize, the expression of most PG genes is up-regulated by light treatments (blue or red), likely to be due to the role of light quality in influencing photosynthesis and leaf senescence. This suggests that specific wavelengths of light may trigger PG formation in maize. In contrast, rice PG genes generally exhibit down-regulation under various stress conditions. For instance, low-temperature treatment significantly reduced the expression of more than half of rice PG genes. This down-regulation contrasts with the responses seen in Arabidopsis and maize.

**Table 1.** Conservativeness of the plastoglobule core protein in model species

*A. thaliana*	Protein name	Full length (amino acid)	*Z. mays* B73	E-value	Match length/full length (amino acid)	Identity (%)	Similarity (%)	*O. sativa* *Japonica*	E-value	Match length/full length (amino acid)	Identity (%)	Similarity (%)	*B. distachyon* BD21-3	E-value	Match length/full length (amino acid)	Identity (%)	Similarity (%)
AT4G04020	Fibrillin 1a/FBN1a	318	Zm00001eb423920	1.00E-110	279/314	59	76	Os09g0133600	2.00E-113	290/319	58	74	Bradi4g08760	6.18E-115	259/325	64	79
AT4G22240	Fibrillin 1b/FBN1b	310	Zm00001eb081140	6.00E-112	243/318	66	83	Os09g0133600	1.00E-113	228/319	70	84	Bradi4g08760	6.02E-110	224/325	70	85
AT2G35490	Fibrillin 2/FBN2	376	Zm00001eb024700	2.00E-109	255/382	69	87	Os10g0575700	1.00E-104	305/374	58	72	Bradi3g34400	8.99E-89	310/352	57	70
AT3G23400	Fibrillin 4/FBN4	284	Zm00001eb114860	2.00E-105	209/272	75	88	Os11g0595200	1.00E-100	200/270	75	87	Bradi4g14630	1.17E-80	190/275	73	86
AT3G58010	Fibrillin 7a/FBN7a	308	Zm00001eb433050	1.00E-115	278/299	56	75	Os04g0665800	6.00E-112	246/301	60	80	Bradi5g25350	4.45E-106	240/299	63	82
AT2G42130	Fibrillin 7b/FBN7b	270	Zm00001eb067150	2.00E-93	222/299	59	76	Os04g0665800	7.00E-93	208/301	61	80	Bradi5g25350	4.59E-75	220/299	60	77
AT2G46910	Fibrillin 8/FBN8	284	Zm00001eb220590	9.00E-112	226/291	69	83	Os10g0509200	5.00E-90	226/286	69	83	Bradi3g30740	2.62E-104	226/280	70	84
AT4G31390	Activity of bc_1_ complex kinase 1/ABC1K1	682	Zm00001eb168450	0	690/680	76	86	Os11g0216300	0	664/675	80	87	Bradi4g22180	0	634/683	82	90
AT1G79600	Activity of bc_1_ complex kinase 3/ABC1K3	711	Zm00001eb347190	0	666/712	72	84	Os05g0323800	0	629/726	75	87	Bradi2g30567	0	589/705	77	89
AT1G71810	Activity of bc_1_ complex kinase 5/ABC1K5	692	Zm00001eb069010	0	446/454	62	78	Os04g0640500	0	672/720	64	79	Bradi5g23450	0	663/705	62	79
AT3G24190	Activity of bc_1_ complex kinase 6/ABC1K6	793	Zm00001eb191420	0	793/775	75	85	Os02g0816600	0	761/784	78	88	Bradi3g55317	0	717/779	81	90
AT3G07700	Activity of bc_1_ complex kinase 7/ABC1K7	695	Zm00001eb225060	0	704/719	72	82	Os09g0250700	0	710/716	71	82	Bradi1g02770	0	710/720	70	81
AT5G05200	Activity of bc_1_ complex kinase 9/ABC1K9	540	Zm00001eb303700	0	537/549	70	84	Os07g0227800	0	514/558	73	86	Bradi2g36950	0	479/549	76	90
AT4G19170	Carotenoid cleavage dioxygenase 4/CCD4	595	Zm00001eb251990	0	524/639	64	79	Os02g0704000	0	524/638	65	79	Bradi3g52680	0	520/647	64	79
AT4G32770	Tocopherol cyclase/VTE1	488	Zm00001eb237270	0	476/439	57	71	Os02g0276500	0	425/470	67	80	Bradi3g10250	0	417/472	67	82
AT1G17420	Lipoxygenase 3/LOX3	919	Zm00001eb005920	0	876/922	65	79	Os03g0179900	0	880/918	69	79	Bradi1g72690	0	888/921	64	78
AT1G54570	Phytyl ester synthase 1/PES1	704	Zm00001eb131690	0	692/701	56	72	Os01g0362100	0	674/698	56	72	Bradi2g12850	0	663/697	57	73
AT3G26840	Phytyl ester synthase 2/PES2	701	Zm00001eb131690	0	703/701	45	63	Os01g0362100	0	706/698	44	62	Bradi4g34846	0	618/673	52	69
AT5G08740	NAD(P)H dehydrogenase C1/NDC1	519	Zm00001eb373110	0	461/872	65	78	Os06g0214900	0	470/548	65	79	Bradi1g45921	0	478/881	63	77
AT1G32220	Flavin reductase-related 1/FDRED1	296	Zm00001eb084520	8.00E-142	259/306	74	86	Os04g0403500	2.00E-144	311/312	65	79	Bradi5g08820	5.89E-141	259/310	72	87
AT2G34460	Flavin reductase-related 2/FDRED2	280	Zm00001eb038110	3.00E-120	269/283	64	77	Os06g0360300	2.00E-56	111/113	79	86	Bradi5g22240	7.68E-110	238/290	72	83
AT3G10130	SOUL Heme-binding protein 4/SOUL4	309	Zm00001eb242810	1.00E-114	253/292	66	78	Os02g0533200	2.00E-103	224/287	66	79	Bradi3g44930	4.16E-96	223/299	67	79
AT1G78140	UbiE methyltransferase-related 1/PGMT1	355	Zm00001eb042280	6.00E-124	326/348	56	74	Os08g0411200	4.00E-118	293/358	59	75	Bradi3g35630	2.03E-122	280/362	63	87
AT2G41040	UbiE methyltransferase-related 2/PGMT2	352	Zm00001eb275150	5.00E-140	293/356	66	79	Os06g0646000	6.00E-139	294/345	63	79	Bradi1g30656	2.00E-136	304/357	62	78
AT1G06690	Aldo/keto reductase/AKRED	377	Zm00001eb117640	0	330/366	76	86	Os07g0143000	0	335/377	80	90	Bradi1g58220	0	358/374	74	86
AT5G41120	Esterase/ELT4	684	Zm00001eb131700	7.00E-163	655/665	40	59	Os01g0361700	9.00E-168	642/664	41	61	Bradi4g34846	0	597/673	52	69
AT4G13200	Plastoglobular protein 18/PG18	185	Zm00001eb426500	8.00E-29	74/158	64	86	Os04g0513000	4.00E-28	73/167	65	87	Bradi5g15520	7.71E-12	43/162	58	79
AT5G13800	Pheophytin pheophorbide hydrolase/PPH	484	Zm00001eb231810	0	392/491	65	81	Os06g0354700	0	449/486	59	75	Bradi1g35780	0	401/492	64	79
AT4G38970	Fructose-bisphosphate aldolase-2/SFBA2	398	Zm00001eb200910	0	397/394	80	89	Os11g0171300	0	398/388	80	87	Bradi4g24367	0	398/389	80	89
AT1G52590	Putative thiol-disulfide oxidoreductase DCC	172	Zm00001eb122820	2.00E-68	154/189	66	82	Os01g0173000	1.00E-67	138/194	71	83	Bradi2g04530	6.19E-54	139/193	69	83
AT3G43540	initiation factor 4F subunit (DUF1350)	373	Zm00001eb313900	6.00E-158	328/414	63	81	Os09g0436900	5.00E-57	133/209	62	81	Bradi4g31070	3.59E-152	326/396	64	82
AT1G28150	Unknown protein, hypothetical protein (U1)	123	Zm00001eb075960	7.00E-25	77/124	62	79	Os04g0528100	4.00E-28	77/127	63	79	Bradi5g16380	3.11E-27	85/129	60	73
AT1G73750	Unknown SAG protein (U2)	452	Zm00001eb042900	5.00E-137	472/524	45	59	Os08g0359300	3.00E-145	475/534	45	60	Bradi3g22010	1.92E-141	498/536	45	60
AT3G27110	Plastoglobular M48 protease/PGM48	344	Zm00001eb127160	2.00E-164	265/335	82	93	Os01g0970700	5.00E-164	282/327	78	90	Bradi2g03717	1.01E-162	274/323	78	92

Homolog protein are identified and extracted from public databases, such as The Plant Proteome Database (PPDB, http://ppdb.tc.cornell.edu/), The Rice Annotation Project Database (RAP-DB, https://rapdb.dna.affrc.go.jp/), MaizeMine (v1.6, https://maizemine.rnet.missouri.edu/), and Phytozome (v13, https://phytozome-next.jgi.doe.gov/info/Bdistachyon_v3_1). Sequence alignments are conducted using the NCBI BLAST tool (https://blast.ncbi.nlm.nih.gov/Blast.cgi).

Field-grown crops are often exposed to multiple environmental stresses simultaneously (e.g. drought and heat). A transcriptional analysis of *Brachypodium* revealed that only a few PG genes responded to single stress treatments. Notably, no PG genes were responsive to drought stress alone. However, when subjected to combined stresses, the transcript levels of some genes exhibited additive or synergistic changes. For example, the expression of *ABC1K6* was repressed by either salinity or heat stress, but its down-regulation was more pronounced under combined stresses. Interestingly, *FBN1*, the key structural gene in PG formation, was the only gene consistently up-regulated across various stress treatments. These transcriptional differences across species suggest that PGs may function in a stress-dependent, species-specific manner.

**Fig. 2.**
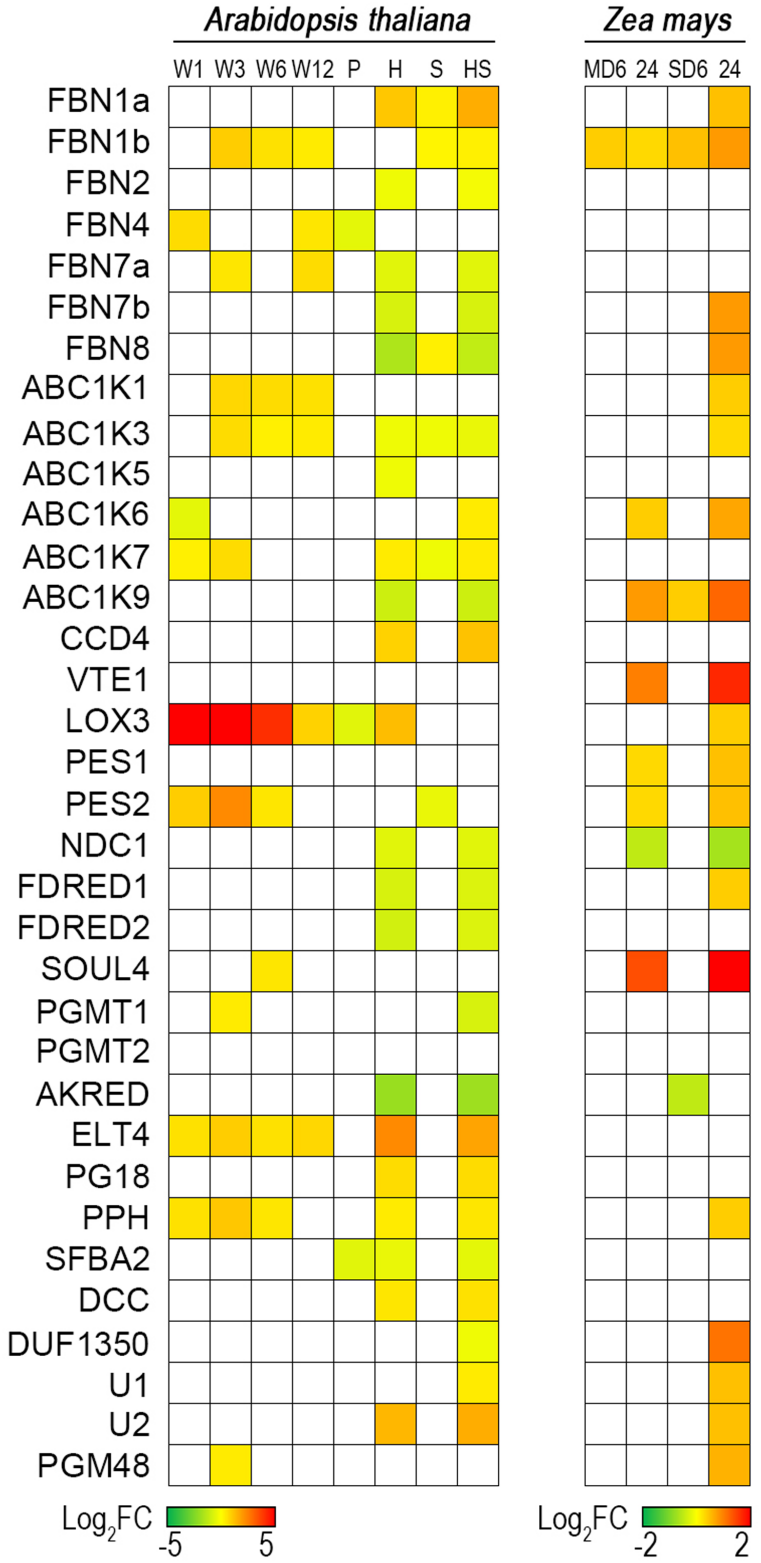
Transcriptional changes of plastoglobule (PG)-localized core genes in Arabidopsis (left panel) and maize (right panel) roots under various abiotic stresses. Data are extracted from and analyzed based on the Expression Altas (https://www.ebi.ac.uk/gxa/home). W1–W12, wounding treatments for 1, 3, 6, and 12 h; P, phosphate limitation; H, heat stress; S, salinity; HS, heat and salinity; MD6 and 24, mild drought treatments for 6 h and 24 h; SD6 and 24, severe drought treatments for 6 h and 24 h. All data are subjected to statistical analysis, and significant differences (*P*adj <0.05) are colored in the heat maps.

Extensive research has focused on characterizing the PG core genes in photosynthetic tissues, yet their dynamics in non-photosynthetic tissues remain largely unexplored. Notably, >60% of maize PG core genes detected in roots were significantly induced by either mild or severe drought conditions. In Arabidopsis roots, PG core genes not only responded to various single stress treatments, such as phosphate limitation, heat stress, and salinity, but they also exhibited strong responses to combined stressors. Furthermore, several of these genes were up-regulated in response to wounding, indicating their roles in root defense mechanisms (Fig. 2; Supplementary Tables S1, S2). Collectively, these findings support the hypothesis that PGs may be produced in roots to enhance plant tolerance to environmental stimuli.

With the rapid advancement of single-cell mass spectrometry-based proteomics, the sensitivity, resolution, and data quality have significantly improved (Bennett *et al.*, 2023), offering new insights into crucial biological processes, including abiotic stress responses. A recent single-cell proteomic study of Arabidopsis leaves demonstrated that severe water deficit significantly increases the relative abundance of several key PG-associated proteins (Fulcher *et al.*, 2024, Preprint). This increase is likely to be due to the stress-induced transcriptional up-regulation of these proteins. Another study on rice confirmed that PG core proteins predominantly accumulate in photosynthetic tissues (Li *et al.*, 2024). Interestingly, in line with transcriptomic data, these proteins were also detected in non-photosynthetic tissues, including roots, reinforcing the idea that PGs may form in the plastids of non-photosynthetic tissues under stress. In these tissues, PGs may act as lipid reserves to support continued organ growth, such as sustaining lateral root elongation under water deficit conditions.

## Future directions and challenges

Plastids, including both classical and specialized types, are interconvertible, membrane-bound organelles found in the cytoplasm of plant and algal cells. They play crucial roles in plant growth, development, and metabolism, including photosynthesis, storage, and the synthesis of essential molecules. Furthermore, plastids act as central hubs for sensing environmental changes, such as light, temperature, water availability, and other abiotic factors, enabling them to modulate cellular processes in response to these cues (Sierra *et al.*, 2023). Recent studies have also identified PG core proteins in non-photosynthetic tissues (e.g. roots), suggesting that PGs may have broader roles beyond photosynthetic tissues. This discovery opens up several key avenues for future research, including: (i) understanding how PG core proteins respond to abiotic stress in non-photosynthetic tissues; (ii) identifying potential tissue- and species-specific PG-associated proteins that remain unknown; and (iii) exploring how PG core proteins function under combined stress conditions. These goals are technically feasible, especially with well-established protocols for extracting high-purity PGs (Shivaiah *et al.*, 2022). To further enhance the purity and homogeneity of PGs, the IPTACT (Isolation of Plastids Tagged in Specific Cell Types) technology could be integrated into existing methodologies. This would allow for more detailed multi-omics studies of PGs across different tissues, cell types, and stress conditions (Boussardon and Keech, 2023).

The role of stress-induced post-translational modifications (PTMs) in PGs remains an open question. Key interactions, such as protein–protein, protein–DNA/RNA, and protein–metabolite interactions, are not yet fully understood. For example, ABC1K proteins are known to interact with and phosphorylate PG-associated proteins (Box 1). Through phosphoproteomic analysis, several conserved phosphorylation sites (p-sites) in PG-associated proteins have been identified (Supplementary Table S5) (Lohscheider *et al.*, 2016). Reversible phosphorylation may act as a switch, regulating PG protein interactions and responses to abiotic stress. However, the regulatory roles of these p-sites remain incompletely validated *in vivo*. There is also a need to further investigate the biochemical and functional implications of PG-localized ABC1Ks, including the synergistic effects of these p-sites on substrate activity and the identification of novel p-sites in proteins and metabolites. Addressing these questions will require continued research and interdisciplinary collaborations.

During developmental transitions, PGs accumulate carotenoids and other prenyl lipids in non-photosynthetic plastids (Liu *et al.*, 2024). The root system plays a crucial role in nutrient and water uptake, and carotenoids are known to be precursors to strigolactones (SLs) and ABA, which regulate root growth. Furthermore, carotenoid-derived bioactive metabolites have been shown to influence root architecture and facilitate communication with the rhizosphere microbiome (Ke *et al.*, 2022). Inhibition of root carotenoid synthesis leads to reduced auxin levels, resulting in shortened primary roots and impaired lateral root formation (Xu *et al.*, 2024). These findings highlight the potentials of PG-localized proteins in carotenoid biosynthesis and metabolism as key players in regulating root architecture and improving plant stress tolerance. This line of research could open up new strategies for enhancing crop resilience by optimizing root systems.

## Concluding remarks

Significant progress has been made in understanding the role of plastid PGs in responses to environmental stimuli, yet many challenges remain before PG proteins can be harnessed to enhance crop tolerance to climate change. For example, the mechanisms by which PG–thylakoid membrane interactions are rebalanced following stress relief are not fully understood. Additionally, the process of vacuole-mediated PG degradation during senescence remains underexplored. This article provides a brief overview of the research achievements related to plastid PGs over the past decade and highlights several unresolved questions. It is hoped that this Viewpoint will inspire further investigation into the multifunctional roles of PGs, unlocking their potential for improving abiotic stress resilience in crops.

## Supplementary data

The following supplementary data are available at *JXB* online.

Table S1. Transcriptional and proteomic changes of PG core genes in Arabidopsis. Data were used to generate Figs 1 and 2.

Table S2. Transcriptional changes of PG core genes in maize. Data were used to generate Figs 1 and 2.

Table S3. Transcriptional and proteomic changes of PG core genes in rice. Data were used to generate Fig. 1.

Table S4. Transcriptional changes of PG core genes in *Brachypodium*. Data were used to generate Fig. 1.

Table S5. Summary of phosphorylated sites of PG core proteins in four model species.

eraf011_suppl_Supplementary_Tables_S1-S5

## Data Availability

The raw data that support the findings of this study are available in the supplementary data.
